# Comparing self- and provider-collected swabbing for HPV DNA testing in female-to-male transgender adult patients: a mixed-methods biobehavioral study protocol

**DOI:** 10.1186/s12879-017-2539-x

**Published:** 2017-06-23

**Authors:** Sari L. Reisner, Madeline B. Deutsch, Sarah M. Peitzmeier, Jaclyn M. White Hughto, Timothy Cavanaugh, Dana J. Pardee, Sarah McLean, Elliot J. Marrow, Matthew J. Mimiaga, Lori Panther, Marcy Gelman, Jamison Green, Jennifer Potter

**Affiliations:** 1 Boston Children’s Hospital, 300 Longwood Avenue, Boston, MA 02215 USA; 2 Harvard Medical School, 25 Shattuck St, Boston, MA 02215 USA; 3 Department of Epidemiology, Harvard T.H. Chan School of Public Health, 677 Huntington Ave, Boston, MA 02215 USA; 4 The Fenway Institute, Fenway Health, 1340 Boylston Street, Boston, MA 02215 USA; 5 Department of Family & Community Medicine, University of California, 2356 Sutter Street, San Francisco, CA 94115 USA; 6 UCSF Center of Excellence for Transgender Health, 2356 Sutter Street, San Francisco, CA 94115 USA; 7 Johns Hopkins School of Public Health, 1340 Boylston Street, Boston, MA 02215 USA; 8 Yale School of Public Health, New Haven, CT USA; 9 Brown University School of Public Health, 121 S Main St, Providence, RI 02903 USA; 10 Alpert Medical School, Brown University, 222 Richmond St, Providence, RI 02903 USA; 11 Center for Health Equity Research (CHER), 121 S Main St, Providence, RI 02903 USA; 12 Beth Israel Deaconess Medical Center, 330 Brookline Ave, Boston, MA 02215 USA; 13 World Professional Association for Transgender Health, 2420 Clover St, Union City, CA 94587 USA

**Keywords:** Transgender, Female-to-male, Cervical cancer, HPV, Sexually transmitted infections (STIs), Screening, Testing, Prevention

## Abstract

**Background:**

Cervical cancer, nearly all cases of which are caused by one of several high-risk strains of the human papillomavirus (hr-HPV), leads to significant morbidity and mortality in individuals with a cervix. Trans masculine (TM) individuals were born with female reproductive organs and identify as male, man, transgender man, or another diverse gender identity different from their female assigned sex at birth. Routine preventive sexual health screening of TM patients is recommended, including screening for cervical cancer and other sexually transmitted infections (STIs); however, as many as one in three TM patients are not up-to-date per recommended U.S. guidelines. Among cisgender (non-transgender) women, self-swab hr.-HPV DNA testing as a primary cervical cancer screening method and self-swab specimen collection for other STIs have high levels of acceptability. No study has yet been conducted to compare the performance and acceptability of self- and provider-collected swabs for hr.-HPV DNA testing and other STIs in TM patients.

**Methods:**

This article describes the study protocol for a mixed-methods biobehavioral investigation enrolling 150 sexually active TM to (1) assess the clinical performance and acceptability of a vaginal self-swab for hr.-HPV DNA testing compared to provider cervical swab and cervical cytology, and (2) gather acceptability data on self-collected specimens for other STIs. Study participation entails a one-time clinical visit at Fenway Health in Boston, MA comprised of informed consent, quantitative assessment, venipuncture for syphilis testing and HIV (Rapid OraQuick) testing, randomization, collection of biological specimens/biomarkers, participant and provider satisfaction survey, and qualitative exit interview. Participants are compensated $100. The primary study outcomes are concordance (kappa statistic) and performance (sensitivity and specificity) of self-collected vaginal HPV DNA specimens vs provider-collected cervical HPV swabs as a gold standard.

**Discussion:**

This study addresses critical gaps in current clinical knowledge of sexual health in TM patients, including comparing alternative strategies for screening and diagnosis of cervical cancer, hr.-HPV, and other STIs. Findings have implications for improving the delivery of sexual health screening to this often overlooked and underserved patient population. Less-invasive patient-centered strategies may also generalize to other at-risk cisgender female populations that face barriers to timely and needed STI and cervical cancer screening.

**Trial registration:**

ClinicalTrials.gov ID: NCT02401867

**Electronic supplementary material:**

The online version of this article (doi:10.1186/s12879-017-2539-x) contains supplementary material, which is available to authorized users.

## Background

Cervical cancer is caused by a sexually transmitted infection with one of several high-risk strains of the human papillomavirus (hr-HPV) in 99.7% of cases [1]. This and other sexually transmitted infections (STIs) lead to significant morbidity and mortality in U.S. cisgender (i.e., non-transgender) females, with 10 million incident STI infections diagnosed annually [2–4]. HPV, a virus passed through direct genital contact, is the most common STI in the U.S. and the cause of virtually all cervical cancers [5]; the estimated prevalence of high-risk oncogenic HPV among cisgender female adolescents and adults is 23%, and approximately 12,500 cases of cervical cancer are diagnosed each year [3]. Papanicolaou (Pap) cytologic testing is recommended to screen for cervical abnormalities in individuals with a cervix ages 21–65 years, with screening every 3 years if Pap test results are normal (“negative” result). HPV co-testing with Pap testing every 5 years is preferred for cisgender females ages 30 years and older. Screening for other STIs (HIV, syphilis, gonorrhea, chlamydia, trichomoniasis, bacterial vaginosis) is also recommended for all patients who engage in high-risk sexual behaviors [2].

Despite myths that masculine-spectrum transgender people (trans masculine people; TM) – individuals assigned a female sex at birth who identify as male, man, trans man, or another diverse gender identity not corresponding to their assigned female sex at birth [6] – are at low risk for STIs, recent research shows that TM patients are at no lower risk for STIs and for cervical abnormalities as compared to cisgender women [7, 8]. Many TM individuals engage in sexual activity with sexual partners of diverse genders (including cisgender men and women, other TM individuals, and transgender women) and have multiple concurrent partners [9]; engage in condomless receptive vaginal and/or anal sex with cisgender men [10]; and demonstrate high rates of STI diagnosis, despite low rates of screening [11].

As the majority (>80%) of TM do not undergo gender affirming genital surgery and therefore retain their cervix, American Congress of Obstetricians and Gynecologists (ACOG) recommend that these TM follow the same cervical cancer screening guidelines as cisgender women [12], and undergo routine screening for other STIs [13]. Despite these recommendations, TM patients appear to be less likely to be current for cervical cancer screening than cisgender women [14, 15]. TM face multilevel barriers to undergoing cervical cancer screening, most notably discrimination by healthcare providers and insurance providers, leading to postponement of care [16]. Structural barriers to regular cervical screening include female-only waiting rooms, woman-centered and heteronormative patient education materials, and language from providers during the screening exam (e.g., “vagina”, “panties”) – all of which fail to recognize and affirm participants’ masculine gender identity [7]. Intrapersonal barriers to testing include a disconnect between birth-assigned sex and self-identified gender; desire to ignore the existence of natal reproductive structures; lack of knowledge that the cervix may be retained after some approaches to hysterectomy; high prevalence of past sexual or emotional trauma; heightened anxiety about having a genital exam; and fear of discrimination on the basis of being transgender [17–23]. Further, TM-specific physiological factors may also impact screening. Long-term testosterone therapy may induce vaginal atrophy and decreased lubrication [21, 23], which may cause physical discomfort with the speculum exam. When TM patients do engage in cervical cancer screening, approximately 11% of Pap specimens in this patient population are found to be inadequate for analysis, as opposed to just 1.3% of tests in comparable cisgender women [7]. There is some evidence of a positive association between increasing rates of inadequate Pap specimens and length of time on testosterone [7]. Patient-centered research is needed to address these structural, intrapersonal, and clinical barriers that are driving low rates of screening in this stigmatized and at-risk patient population.

Self-collected hr.-HPV DNA testing as a primary cervical cancer screening technique [24–26] and self-swabs for STI testing [27–33] have been tested in hard-to-reach non-transgender female patients who are unlikely to engage in provider-administered screening. This methodology involves the use of a cotton- or polyester-tipped swab by a patient to self-collect a sample from the vaginal canal, without the use of a speculum. High-risk HPV DNA testing as a primary screening strategy for cervical cancer has been found in one study to have superior sensitivity compared to the current standard of care of cytologic screening alone or a hybrid screening strategy of cytology/h-HPV co-testing [26]. Additional studies have also found self-collected vaginal specimens for primary hr.-HPV screening to be more acceptable than cervical cytologic screening among cisgender females due to the less invasive nature of self-collection, which in turn lead to increased adherence to screening recommendations in underscreened populations [24, 25, 34]. This modality also represents a potential approach to bridging barriers to screening among TM populations, as it minimizes or eliminates barriers such as pain during speculum exam, prevalent cytological inadequacy, and fear of negative interactions with providers during a pelvic exam. No studies have yet been conducted administering these tests and exploring their acceptability and clinical performance among TM patients.

The aims of this study were to 1) quantitatively and qualitatively assess the acceptability and clinical performance of a vaginal self-swab for hr.-HPV DNA testing compared to provider hr.-HPV and cervical cytologic cotesting among sexually active TM, and 2) investigate the prevalence of other STIs among sexually active TM. This study addresses critical gaps in current clinical knowledge of sexual health in TM patients, including comparing alternative strategies for prevention, screening, and diagnosis. We hypothesized that our findings would improve patient care, including attitudes about and comfort with screening, and delivery of preventive sexual health screening in this overlooked and underserved patient population, including potentially less-invasive, alternative screening strategies for TM patients.

## Methods & design

### Design

This biobehavioral mixed-methods study examined preventive sexual health screening in 150 TM patients in Boston, MA. Community-based participatory research principles (CBPR) [35, 36] were employed throughout the study. A 10-member project-specific Task Force comprised of TM patients, providers, and stakeholders was established. The Task Force was comprised of 60% TM individuals who were recruited via referrals from Fenway staff and other TM community members. The Task Force met every other month (6 times per year) to provide input on all aspects of study design and conduct. Members were compensated $50 per meeting for their time. The Task Force developed a mission statement and bylaws that emphasized reciprocal relationships, co-learning, partnership, trust, transparency, and honesty with all members of the study team, including investigators, staff, patients, providers, and stakeholders.

### Setting

This study was conducted at The Fenway Institute at Fenway Health in Boston, MA. As of 2013, Fenway Health had approximately 650 TM patients actively engaged in medical care; by 2016 that number had increased to over 950 TM patients. All data were collected between April 2015 and September 2016. All study procedures were approved by the Fenway Health Institutional Review Board (FWA00000145).

### Characteristics of participants

Individuals were eligible to participate in the one-time clinical study visit if they met the following criteria: 1) Ages 21–64 years (consistent with current cervical cancer screening recommendation); 2) Assigned a female sex at birth and now identifies a man, trans man, male, trans masculine, FTM (female-to-male), transgender, genderqueer/non-binary, transsexual, and/or another diverse transgender identity; 3) Have a cervix; 4) Sexually active in the past 36 months (with partner(s) of any gender); 5) Able to speak and understand English; and 6) Willing and able to provide informed consent.

#### Note on HPV vaccination

The research team considered excluding individuals who had received the HPV vaccine, but decided to enroll individuals regardless of HPV vaccination status for the following reasons: 1) current cervical cancer screening guidelines are identical for HPV vaccinated and unvaccinated individuals; 2) in our formative qualitative work, 62% reported never having received any dose of the HPV vaccination (unpublished data); 3) some TM individuals who are vaccinated will not have been vaccinated until after sexual debut and may have already been exposed to HPV; 4) there is a substantial risk of recall bias when relying on HPV vaccination self-report, especially series completion; 5) given the relatively recent introduction of routine HPV vaccination recommendations, it is far less likely for our subjects over the age of 30 years to have received a vaccination series; and 6) no research has reported prevalence of HPV vaccination in TM individuals, thus collecting information about HPV vaccination and number of doses completed would be a valuable aspect of the study.

#### Note on enrolling individuals who are up-to-date on cervical cancer screening

The research team considered whether to enroll participants self-reporting a Pap test in the prior 3 years, as this would in reality represent over-screening. Ultimately, the research team decided to enroll individuals regardless of self-report of last screening date or result, as self-report of last date of cervical cancer screening has been shown to be an inaccurate measure of actual screening utilization [37–39]. Participants were educated on the potential risks associated with over-screening (i.e., potentially unnecessary further testing such as colposcopy) during the informed consent process.

### Study procedures

#### Recruitment

Multiple convenience and referral-based sampling techniques were used by study staff to identify and recruit potentially eligible participants from the catchment area of Fenway Health’s five clinics. Purposive sampling techniques were also used to ensure a diverse study sample with regards to age, race, and ethnicity. All recruitment was conducted by study staff who are also members of the TM community or allies.

Active recruitment was conducted by working with the Fenway Health data informatics team to identify patients coming in for routine care who were potentially eligible for the study through the electronic medical record. Staff asked providers to provide information about the study to patients identified as potential participants, as well as a card containing contact information for the study.

Potential participants were also recruited passively via the posting of study flyers at Fenway Health locations, as well as at community spaces frequented by TM individuals. Recruitment advertisements were posted to online platforms such as the Fenway research website, MyFenway and MyBorum (Fenway Health patient portals), Facebook, transgender community list serves, paid advertisements on Apps such as Scruff and FetLife, and in other local media such as the 2016 Boston Pride Guide. A study-specific website and a Facebook page were also developed to aid in recruitment and dissemination of information about the study.

#### Screening for eligibility

Individuals interested in participating consented to eligibility screening prior to enrollment. All study visits occurred within 1 month of screening. When scheduling the study visit, eligible individuals were given the option of seeing either a cisgender male or cisgender female study provider, and told that they had the option of bringing a support person into the exam room during the Pap test to help the participant feel more comfortable.

#### Clinical visit procedures

**Fig. 1:**
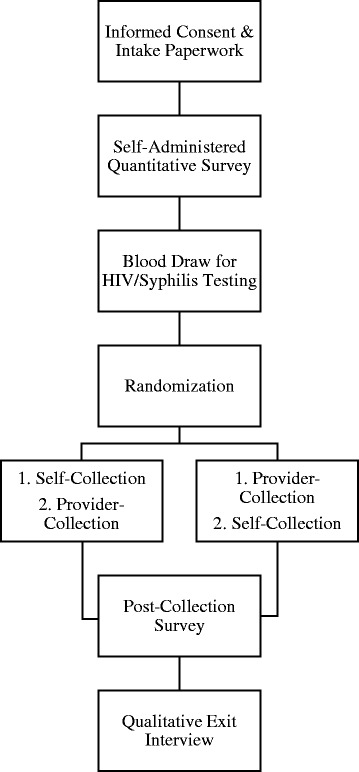
Diagram of study visit flow

See Fig. 1 for an overview of the one-time clinical visit procedures. All visits consist of an/a: (1) Informed Consent Process; (2) Quantitative Assessment; (3) Venipuncture for Syphilis Testing and HIV (Rapid OraQuick) Testing; (4) Randomization; (5) Collection of Biological Specimens/Biomarkers; (6) Participant and Provider Satisfaction Survey; and (7) Qualitative Exit Interview. All study visits were conducted in a private and secure location at Fenway Health. Study visits lasted approximately 3 to 4 h. Participants were compensated $100 for participating in the form of a pre-paid American Express gift card.

1. *Informed Consent & Intake Paperwork* – Written informed consent was obtained from each participant prior to the initiation of study procedures or assessments.
2. *Self-Administered Quantitative Survey* – All participants completed a quantitative assessment via computer-assisted interview technology (CASI) via a touch-screen iPad in self-administered format to increase the accurate reporting of sensitive questions (e.g., sexual behaviors, STI history) [40, 41]. To minimize cultural bias and maximize appropriateness and comparability to other research studies, we selected measures that were previously tested with diverse populations (including transgender people) whenever possible. See Table 1 for a full list of measures included in the quantitative assessment.
3. *Blood Draw for HIV & Syphilis Testing* – A venipuncture was performed by study staff to screen for syphilis and HIV. One 2 mL tube was drawn for a Rapid Plasma Reagin (RPR) (Quest Diagnostics, Marlborough, MA, USA) with reflex to titer and confirmatory test. A second tube was used to collect a small blood sample to conduct a rapid HIV test using the FDA-approved OraQuick® ADVANCE™ HIV-1/2 Antibody Test [sensitivity: 99.6% (98.5–99.9); specificity: 100% (99.7–100)] (OraSure Technologies Inc., Bethlehem, PA, USA). All counseling and testing procedures were consistent with HIV testing and counseling requirements set forth by the Massachusetts Department of Public Health. There were three scenarios in which a participant did not need to undergo HIV testing: 1) the participant did not consent to the test, 2) the participant was HIV-infected and can provide documentation, and 3) the participant had been tested for HIV in the last 2 months and could provide sufficient documentation of their HIV-negative status (e.g., letter from the participant’s provider/testing counselor, lab report).
4. *Randomization* – To guard against potential specimen collection ordering effects, participants were randomized to do either self- or provider-collection first. Participants did not have an option as to which order specimen collection was done. A randomization table was generated using Statistical Analysis Software (SAS) Version 9.4.
5. *Collection of Biological Specimens* – See Table 2 for a listing of all specimens collected during the one-time clinical visit and mode of collection (i.e., self- or provider-collected). See Tables 3 and 4 for collection materials and analysis of self-collected and provider-collected specimens, respectively. All specimen collection occurred in a private exam room at Fenway Health. All tests were performed by Quest Diagnostics (Marlborough, MA, USA) for analysis, with the exception of trichomoniasis and bacterial vaginosis rapid tests, which were performed via OSOM® Rapid Test (Sekisui Diagnostics LLC, San Diego, CA, USA).
  - Self-Collection Procedures. Participants self-collected three swabs: 1) vaginal swabs hr.-HPV DNA, 2) vaginal swab for gonorrhea/chlamydia (GC/CT) testing, and 3) a rectal swab for GC/CT testing. Participants were instructed by study staff on specimen self-collection methods, and were provided an instruction sheet for reference during self-collection (Additional file 1). Testing swabs and collection tubes were color coded to prevent confusion. Study staff remained available for assistance during the self-collection procedure. Once self-collection was complete, participants delivered the specimens to study staff, who then labeled and packaged the samples per performing laboratory guidelines.
  - Provider-Collection Procedures. Multiple providers (two cisgender male, three cisgender female) were trained in specimen collection according to study protocol. The exam and collection process were standardized. Providers conducted a brief pre-exam assessment and medical history using a script adapted from the Center for Disease Control and Prevention’s *A Guide to Taking a Sexual Health History* [42] (Additional file 2). In order to insure fidelity, evaluate consistency between the actual occurrences of the visit and what was documented by the study provider, and identify any inter-provider variability that could confound results, some provider sessions were audio recorded; each study clinician had a minimum of 10 sessions recorded the recordings were reviewed by an off-site expert. The provider collected a pharyngeal swab for GC/CT testing.
  Participants were given several procedural options to help reduce exam anxiety, such as self-insertion of the speculum with verbal guidance by the provider, lying feet flat on the exam table instead of using stirrups, and examining the various instruments to be used prior to the exam. The provider then inserted an appropriately sized speculum using water-soluble lubricant applied along the sides of the speculum, avoiding the tip [43]; the use of lubricant in this population is important given the high prevalence of anxiety and vaginal atrophy; the use of manufacturer-specified lubricant does not interfere with cytologic testing essential and appropriate [44]. The provider collected four vaginal swabs for 1) hr.-HPV DNA testing, 2) GC/CT testing, 3) trichomoniasis vaginalis, and 4) bacterial vaginosis testing. (Note: the provider-collected vaginal hr.-HPV DNA swab was added after participant 95). The provider then collected a cervical specimen (Pap) for cervical cytology and hr.-HPV DNA testing. The provider delivered specimens to study staff, who labeled and packaged the samples per laboratory guidelines.
6. *Post-Collection Survey* - Immediately following collection of biological specimens, patients and providers each separately completed a nine-question questionnaire (Additional file 3) to gather dyadic information on patient/provider comfort and satisfaction.
7. *Qualitative Exit Interview* **-** At the end of the study visit, all 150 TM participants completed a brief, semi-structured qualitative exit interview lasting approximately 30–45 min. Interviews were audio recorded and transcribed verbatim.

**Table 1:** Information queried via quantitative assessment

Section Topic Area	Specific Content
Socio-demographics	Age; Race/Ethnicity; Education; Employment status; Income; Relationship status; Children; Housing stability; Zip code; Cross streets
Transgender History & Gender Affirmation	Sex assigned at birth; Gender identity; Pronouns; Childhood gender behavior/feelings; Ages of transgender first awareness, gender affirmation, disclosure; Legal gender affirmation status (i.e., legal documents); Internalized stigma; Physical gender affirmation status (i.e., binding, testosterone, surgery); Access to gender-affirming care
General Healthcare Access (for routine care not related to transition)	Insurance; Barriers to accessing care; Healthcare satisfaction; Healthcare avoidance; Experiences of discrimination in healthcare; Anticipated stigma in healthcare
General Sexual Health	Age of menarche; Gender(s) of sexual partners (lifetime); Age at first intercourse; History of contraception, pregnancy, and childbirth; Parenting desires; Beliefs about cervical Pap tests; Pap testing history; HPV vaccine and testing history; HPV risk beliefs; HIV testing history; PrEP knowledge and use; STI testing, diagnosis, treatment, and partner notification history; Libido and sexual satisfaction; STI knowledge and beliefs; Anticipated acceptability of and comfort with self- and provider-collection methods
Sexual Risk Activity	Sexual orientation; Gender(s) of sexual partners (past 36 months); Unprotected sexual contact (past 36 months); Partner-level sexual activity (3 most recent partners in past 12 months) ➔ Gender of partner; Relationship type; Sexual activities (e.g., oral-genital performed/received, receptive and/or insertive vaginal or anal sex) and barrier use frequency; Partner HIV/STI status
Trauma, Victimization & Resilience	Childhood abuse/trauma (prior to 18 years of age); Adult abuse/trauma; Intimate Partner Violence (lifetime and past 12 months); Victimization and attribution; Physical symptoms due to victimization; Stress recovery/Resilience; Social support
Mental Health & Substance Use	Mental health treatment (lifetime and current); Depression (CESD-10); Anxiety (BSI-18); Self- and community-acceptance; Suicide attempts (lifetime and past 12 months); Non-lethal self-injury (lifetime and past 12 months); Alcohol, tobacco, and other drug use (past 6 months)
Other	Cancer history (self and family); BMI; Weight description; Weight management; Body consciousness; Nutrition; Exercise

**Table 2:** Specimens collected during the one-time clinical visit

Self-Collected	(2) Vaginal swabs	1. Vaginal HPV 2. Vaginal GC/CT
	(1) Rectal swab	1. Rectal GC/CT
Provider Collected	(1) Pharyngeal swab	1. Pharyngeal GC/CT
	(4) Vaginal swabs	1. Vaginal HPV 2. Vaginal GC/CT 3. Trichomonas Vaginalis (Analyzed via Rapid Test) 4. Vaginal Bacterial Vaginosis (Analyzed via Rapid Test)
	(1) Cervical swab	1. Cervical cytology and HPV specimen (Pap)

**Table 3:** Collection materials and analysis of self-collected specimens

Specimen	Collection Materials	Lab Analysis (Quest Diagnostics)
Vaginal HPV DNA	Polyester Swab (Puritan) ThinPrep Solution (Cytyc®)	DNA Hybridization Assay via Digene probe (HPV Types 16, 18, 31, 33, 35, 39, 45, 51, 52, 56, 58, 59, 68)
Vaginal GC/CT	Aptima Unisex Swab Specimen Collection Kit for Female Endocervical and Male Urethral Swab Specimens	
Rectal GC/CT	Aptima Unisex Swab Specimen Collection Kit for Female Endocervical and Male Urethral Swab Specimens	

Additional supplies provided to each participant: Hand mirror, latex gloves, written self-collection instructions

Collection kits for each specimen were color coded and numbered to match to the respective instruction sheet and to mitigate errors in sample packaging

**Table 4:** Collection materials and analysis of provider-collected specimens

Specimen	Collection Materials	Lab Analysis (Quest Diagnostics)
Pharyngeal GC	Aptima Unisex Swab Specimen Collection Kit for Female Endocervical and Male Urethral Swab Specimens	
Vaginal GC/CT	Aptima Unisex Swab Specimen Collection Kit for Female Endocervical and Male Urethral Swab Specimens	
Vaginal Trichomonisas	OSOM Trichomonas Rapid Test Kit, Sterile Swab	
Vaginal BV	OSOM Bvblue Rapid Test Kit, Sterile Swab	
Vaginal HPV DNA	Polyester Swab (Puritan) Thinprep Solution (Cytyc®)	DNA Hybridization Assay via Digene probe (HPV Types 16, 18, 31, 33, 35, 39, 45, 51, 52, 56, 58, 59, 68)
Cervical HPV DNA/mRNA & Cytology	Medscand® Pap-Perfect® Spatula and Cytobrush Plus (Cooper Surgical) Cytyc® ThinPrep® solution	

For collection of all vaginal samples: Welch-Allyn speculum, halogen plug-in speculum light, water-soluble lubricant, large cotton swab to remove excess lubricant at time of speculum removal

Interviews were conducted to qualitatively understand acceptability of different cervical cancer and STI screening technologies. Participants answered questions about comparative acceptability of self-collected vaginal swab for HPV vs. provider-collected vaginal swab for hr-HPV vs. cervical cytology alone, and self-vs. provider-collected swabs for other STIs. In order to evaluate acceptability and feasibility, patients were also queried on the perceived difficulty of self-swabs, in line with methods in prior qualitative studies [45]. Participants also answered questions on preference for and acceptability of different clinical algorithms in screening taking into consideration potential risks of over-screening (and need for colposcopy) when using a hr-HPV-only or hr-HPV-triage approach [26]. Lastly participants were asked general questions relating to study procedures.

A subsample of 50 participants were purposively sampled to undergo a more in-depth interview (additional 30 minute interview, additional $10 incentive). Participants were invited to undergo the longer interview to maximize diversity in demographics (age, gender identity, race/ethnicity, recent sexual behavior) as well as to maximize diversity of opinion as expressed in the first part of the interview (e.g., opinion on acceptability of the self-swabs). The in-depth qualitative interview covered the previously described exit interview topics as well as additional domains to inform future clinical practice and research: 1) narrative history of preventive sexual health screening; 2) barriers and facilitators to screening utilization guided by Health Belief Model constructs [46]; 3) past and current experiences of stigma in accessing preventative sexual health screening guided by the “hidden distress” model [47], including felt and enacted stigma; 4) acceptability of sexual health screening questions to inform further development of a clinical tool that can be used to standardize sexual and behavioral risk screening; 5) acceptability of biomedical prevention strategies in high-risk TMs, such as Pre-Exposure Prophylaxis (PrEP) for HIV prevention.

### Statistical analysis

The primary outcome variables of the study are concordance (kappa statistic) and performance (sensitivity and specificity) of self-collected vaginal HPV DNA specimens vs provider-collected cervical HPV swabs as a gold standard. McNemar’s test is a two-sample test for binomial proportions for matched-pair data [48]. An adjusted McNemar test [49] will be used to compare the proportions of hr.-HPV DNA-positive results between samples while accounting for the correlation of multiple samples within subjects [50]. This analytic approach has been used previously in hr.-HPV research assessing concordance of self/provider swabs [51]. The concordance of hr.-HPV DNA detection between sampling modalities will be assessed using an unweighted Kappa (K) statistic to determine the percentage agreement beyond that expected by chance [52], and jackknife estimation will be used to compute the variance while accounting for correlation within subjects. Sensitivity, the probability of a positive test given that the individual has the condition, will be calculated as *sensitivity = true positive/(true positive + false negative)*. Specificity, the probability of correcting detecting individuals without a condition, will be estimated as *specificity = true negative/(true negative + false positive)*.

#### Power and sample size

Power for the adjusted McNemar’s test is dependent on multiple parameters, including hr.-HPV prevalence in our sample and the difference in detection rates between self/provider sampling methods.

1. *HPV prevalence estimates* - Overall, prevalence of hr.-HPV in the U.S. is 42.5% in females ages 14–59 years captured via self-collection of cervicovaginal swab [lowest among age 50–59 (23.5%) and highest among age 20–24 (43.3%)] [4]. In a meta-analysis of self-swab studies, average HPV detection rate across 10 studies was 24.1% (95% CI = 22.8–25.5%) with self-sampling, and 24.8% (95% CI = 23.4–26.1%) with provider sampling [53]. There are no hr.-HPV prevalence studies of TM individuals. Research suggests lower prevalence of hr.-HPV among women who have sex with women (WSW) compared to behaviorally heterosexual women. Among 133 WSW, detectable HPV DNA was 30%, and 19% among WSW who did not have a lifetime history of sex with men [54]. Given uncertainty around participants’ sexual behavior, we therefore conservatively powered our study with an estimated hr.-HPV prevalence of 15%.
2. *Concordance of vaginal self-swab and provider cervical swab for hr.-HPV DNA testing* – Comparing the concordance of the hr.-HPV DNA-positive results to the cervical provider swab hr.-HPV DNA test results (“gold standard” reference) using the McNemar’s test, a two-sample test for binomial proportions for matched-pair data. The null hypothesis is that the sensitivities of swab 1 (self-collected frontal/vaginal) and swab 2 (provider-collected cervical specimen) are equivalent. Assuming a 15% hr.-HPV prevalence via vaginal self-swab, we selected a sample size of 150 to detect a 15% or more discordance between the two sampling approaches (and achieve 80% power at the 0.05 alpha-level. A meta-analysis of hr.-HPV self-swab studies found the range of absolute differences in detection rates between swab sampling methods (self vaginal, provider cervical) was most often between 0.14%–22.2% (median 19%) [53].

### Qualitative analysis

Qualitative data will be analyzed using a combination of traditional content analysis [55] and techniques borrowed from grounded theory [56]. Transcripts will first be reviewed for errors and omissions, and identifying information will be redacted. Study staff will then open code a series of transcripts and group the open codes into a codebook according to themes. A team of two analysts will then individually code the transcripts using Dedoose software [57]. The analysts will meet regularly throughout the coding process to discuss emerging themes and add additional codes as needed. Coded data will be analyzed through ongoing discussion between coders and investigators to allow for interconnections between research questions, coding transcripts, and raw data [56].

## Discussion

This study addresses critical gaps in current clinical knowledge of sexual health in TM individuals. We are the first to our knowledge to describe rates of cervical high-risk HPV DNA infection and of STIs in a study focused specifically on TM individuals. We are also the first to our knowledge to examine the role and performance of self-collected hr.-HPV DNA testing as a primary cervical cancer screening strategy in TM individuals. Given evidence that self-collected hr.-HPV DNA testing may improve screening rates in underscreened populations of cisgender women [34], this screening modality could prove critical in addressing screening disparities for TM individuals, if the clinical performance of the test is adequate. In addition to self-collected hr.-HPV DNA testing, findings may also support alternative patient-centered screening strategies for other STIs in TM individuals. This community-engaged, patient-centered research will allow providers to become better informed on approaches to caring for TM individuals, and has the potential to improve uptake of sexual health screening in this marginalized population. Findings have implications for delivery of preventive sexual healthcare screening in TM patients and may generalize to other at-risk cisgender female populations that face barriers to timely and needed STI and cervical cancer screening. The study can inform a future randomized controlled trial (RCT) of an intervention to test the efficacy of self-collected swabbing for hr.-HPV DNA testing and other STIs vs. usual clinical care to improve preventive sexual health screening uptake in TM patients.

## Additional files

Additional file 1: Diagram & Definition of Terms. (PDF 311 kb)

Additional file 2: Provider Assessment and Interaction Tool. (DOCX 34 kb)

Additional file 3: Patient Satisfaction Survey. (DOC 48 kb)

## Abbreviations

CT: Chlamydia
FTM: Female-to-Male
GC: Gonorrhea
HPV: Human papillomavirus
Pap test: Papanicolaou test
STI: Sexually transmitted infection
TM: Trans masculine
WSW: Women who have sex with women

## References

[1] Walboomers JM, Jacobs MV, Manos MM, Bosch FX, Kummer JA, Shah KV, Snijders PJ, Peto J, Meijer CJ, Munoz N (1999). Human papillomavirus is a necessary cause of invasive cervical cancer worldwide. J Pathol.

[2] Center for Disease Control and Prevention (CDC). Sexually transmitted diseases treatment guidelines. 2010. https://www.cdc.gov/std/treatment/2010/default.htm. Accessed 15 Feb 2017.

[3] Center for Disease Control and Prevention (CDC). Other Sexually Transmitted Diseases. 2011. https://www.cdc.gov/std/stats11/other.htm. Accessed 15 Feb 2017.

[4] Hariri S, Unger ER, Sternberg M, Dunne EF, Swan D, Patel S, Markowitz LE (2011). Prevalence of genital human papillomavirus among females in the United States, the National Health and Nutrition Examination Survey, 2003-2006. J Infect Dis.

[5] National Cancer Institute (NCI). Fact sheet: HPV and Cancer. 2013. http://www.cancer.gov/cancertopics/factsheet/Risk/HPV. Accessed 16 Feb 2017.

[6] Institute of Medicine (IOM) (2011). The health of lesbian, gay, bisexual, and transgender people: building a Foundation for Better Understanding.

[7] Peitzmeier SM, Reisner SL, Harigopal P, Potter J (2014). Female-to-male patients have high prevalence of unsatisfactory Paps compared to non-transgender females: implications for cervical cancer screening. J Gen Intern Med.

[8] Reisner SL, Murchison GR (2016). A global research synthesis of HIV and STI biobehavioural risks in female-to-male transgender adults. Glob Public Health.

[9] Bauer GR, Travers R, Scanlon K, Coleman TA (2012). High heterogeneity of HIV-related sexual risk among transgender people in Ontario, Canada: a province-wide respondent-driven sampling survey. BMC Public Health.

[10] Schulden JD, Song B, Barros A, Mares-DelGrasso A, Martin CW, Ramirez R, Smith LC, Wheeler DP, Oster AM, Sullivan PS (2008). Rapid HIV testing in transgender communities by community-based organizations in three cities. Public Health Rep.

[11] Reisner SL, Perkovich B, Mimiaga MJ (2010). A mixed methods study of the sexual health needs of New England transmen who have sex with nontransgender men. AIDS Patient Care STDs.

[12] American College of Obstetricians and Gynecologists (ACOG) (2011). Committee opinion no. 512: health care for transgender individuals. Obstet Gynecol.

[13] UCSF Department of Family and Community Medicine. Primary care protocol for transgender patient care. 2011. http://transhealth.ucsf.edu/trans?page=protocol-screening#S1X. Accessed 15 Feb 2017.

[14] Peitzmeier SM, Khullar K, Reisner SL, Potter J (2014). Pap test use is lower among female-to-male patients than non-transgender women. Am J Prev Med.

[15] Dutton L, Koenig K, Fennie K (2008). Gynecologic care of the female-to-male transgender man. J Midwifery Womens Health.

[16] Reisner SL, Conron K, Scout N, Mimiaga MJ, Haneuse S, Austin SB (2014). Comparing in-person and online survey respondents in the U.S. National Transgender Discrimination Survey: implications for transgender Health Research. LGBT Health.

[17] Driak D, Samudovsky M (2005). Could a man be affected with carcinoma of cervix?--the first case of cervical carcinoma in trans-sexual person (FtM)--case report. Acta medica (Hradec Kralove)/Universitas Carolina. Facultas Medica Hradec Kralove.

[18] Feldman JL, Ettner R, Monstrey S, Eyler E (2007). Preventive care of the transgendered patient: an evidence-based approach. Principles of transgender medicine and surgery.

[19] Kaufman R, Makadon H, Mayer KH, Potter J, Goldhammer H (2008). Introduction to transgender identity and health. The Fenway Guide to lesbian, gay, bisexual, and transgender health.

[20] Kenagy GP (2005). Transgender health: findings from two needs assessment studies in Philadelphia. Health Soc Work.

[21] O'Hanlan KA, Dibble SL, Young-Spint M (2007). Total laparoscopic hysterectomy for female-to-male transsexuals. Obstet Gynecol.

[22] Rachlin K, Green J, Lombardi E (2008). Utilization of health care among female-to-male transgender individuals in the United States. J Homosex.

[23] van Trotsenburg MAA (2009). Gynecological aspects of transgender healthcare. Int J Transgenderism.

[24] Dannecker C, Siebert U, Thaler CJ, Kiermeir D, Hepp H, Hillemanns P (2004). Primary cervical cancer screening by self-sampling of human papillomavirus DNA in internal medicine outpatient clinics. Ann Oncol.

[25] Nobbenhuis MA, Helmerhorst TJ, van den Brule AJ, Rozendaal L, Jaspars LH, Voorhorst FJ, Verheijen RH, Meijer CJ (2002). Primary screening for high risk HPV by home obtained cervicovaginal lavage is an alternative screening tool for unscreened women. J Clin Pathol.

[26] Wright TC, Stoler MH, Behrens CM, Sharma A, Zhang G, Wright TL (2015). Primary cervical cancer screening with human papillomavirus: end of study results from the ATHENA study using HPV as the first-line screening test. Gynecol Oncol.

[27] Chernesky MA, Hook EW, Martin DH, Lane J, Johnson R, Jordan JA, Fuller D, Willis DE, Fine PM, Janda WM (2005). Women find it easy and prefer to collect their own vaginal swabs to diagnose Chlamydia trachomatis or Neisseria gonorrhoeae infections. Sex Transm Dis.

[28] Garland SM, Tabrizi SN (2004). Diagnosis of sexually transmitted infections (STI) using self-collected non-invasive specimens. Sex Health.

[29] Hobbs MM, van der Pol B, Totten P, Gaydos CA, Wald A, Warren T, Winer RL, Cook RL, Deal CD, Rogers ME (2008). From the NIH: proceedings of a workshop on the importance of self-obtained vaginal specimens for detection of sexually transmitted infections. Sex Transm Dis.

[30] Holland-Hall CM, Wiesenfeld HC, Murray PJ (2002). Self-collected vaginal swabs for the detection of multiple sexually transmitted infections in adolescent girls. J Pediatr Adolesc Gynecol.

[31] Huppert JS, Hesse EA, Bernard MA, Xiao Y, Huang B, Gaydos CA, Kahn JA (2011). Acceptability of self-testing for trichomoniasis increases with experience. Sex Transm Infect.

[32] Knox J, Tabrizi SN, Miller P, Petoumenos K, Law M, Chen S, Garland SM (2002). Evaluation of self-collected samples in contrast to practitioner-collected samples for detection of Chlamydia trachomatis, Neisseria gonorrhoeae, and Trichomonas vaginalis by polymerase chain reaction among women living in remote areas. Sex Transm Dis.

[33] Stewart CM, Schoeman SA, Booth RA, Smith SD, Wilcox MH, Wilson JD (2012). Assessment of self taken swabs versus clinician taken swab cultures for diagnosing gonorrhoea in women: single centre, diagnostic accuracy study. BMJ.

[34] Gok M, van Kemenade FJ, Heideman DA, Berkhof J, Rozendaal L, Spruyt JW, Belien JA, Babovic M, Snijders PJ, Meijer CJ (2012). Experience with high-risk human papillomavirus testing on vaginal brush-based self-samples of non-attendees of the cervical screening program. Int J Cancer.

[35] Clements-Nolle K, Bachrach A (2002). Community based participatory research with a hidden population: the transgender community health project. In Minkler M & Wallerstein N, editors. Community-based participatory research for health.

[36] Leung MW, Yen IH, Minkler M (2004). Community based participatory research: a promising approach for increasing epidemiology's relevance in the 21st century. Int J Epidemiol.

[37] McPhee SJ, Nguyen TT, Shema SJ, Nguyen B, Somkin C, Vo P, Pasick R (2002). Validation of recall of breast and cervical cancer screening by women in an ethnically diverse population. Prev Med.

[38] Pizarro J, Schneider TR, Salovey P (2002). A source of error in self-reports of pap test utilization. J Community Health.

[39] Suarez L, Goldman DA, Weiss NS (1995). Validity of pap smear and mammogram self-reports in a low-income Hispanic population. Am J Prev Med.

[40] Boekeloo BO, Schiavo L, Rabin DL, Conlon RT, Jordan CS, Mundt DJ (1994). Self-reports of HIV risk factors by patients at a sexually transmitted disease clinic: audio vs written questionnaires. Am J Public Health.

[41] Mackenzie SL, Kurth AE, Spielberg F, Severynen A, Malotte CK, St Lawrence J, Fortenberry JD (2007). Patient and staff perspectives on the use of a computer counseling tool for HIV and sexually transmitted infection risk reduction. J Adolesc Health.

[42] Center for Disease Control and Prevention (CDC): A Guide to Taking a Sexual Health History, 2005. https://www.cdc.gov/std/treatment/sexualhistory.pdf. Accessed 15 Feb 2017.

[43] Hologic, Inc (2012). Re: lubricant use during pap sample collection.

[44] Hologic, Inc (2012). ThinPrep pap test, application FAQs.

[45] Igidbashian S, Boveri S, Spolti N, Radice D, Sandri MT, Sideri M (2011). Self-collected human papillomavirus testing acceptability: comparison of two self-sampling modalities. J Women's Health (Larchmt).

[46] Rosenstock IM, Glanz K, Lewis FM, Rimer BK (1990). The health belief model: explaining health behavior through expectancies. Health behavior and health education: theory, research, and practice.

[47] Scambler G (2004). Re-framing stigma: felt and enacted stigma and challenges to the sociology of chronic and disabling conditions. Soc Theor Health.

[48] Rosner B (2006). Fundamentals of biostatistics.

[49] McNemar Q (1947). Note on the sampling error of the difference between correlated proportions or percentages. Psychometrika.

[50] Durkalski VL, Palesch YY, Lipsitz SR, Rust PF (2003). Analysis of clustered matched-pair data. Stat Med.

[51] Winer RL, Feng Q, Hughes JP, Yu M, Kiviat NB, O'Reilly S, Koutsky LA (2007). Concordance of self-collected and clinician-collected swab samples for detecting human papillomavirus DNA in women 18 to 32 years of age. Sex Transm Dis.

[52] Fleiss JH (1981). The measurement of interrater agreement. Statistical methods for rates and proportions.

[53] Petignat P, Faltin DL, Bruchim I, Tramer MR, Franco EL, Coutlee F (2007). Are self-collected samples comparable to physician-collected cervical specimens for human papillomavirus DNA testing? A systematic review and meta-analysis. Gynecol Oncol.

[54] Marrazzo JM (2000). Genital human papillomavirus infection in women who have sex with women: a concern for patients and providers. AIDS Patient Care STDs.

[55] Betancourt TS, Speelman L, Onyango G, Bolton P (2009). A qualitative study of mental health problems among children displaced by war in northern Uganda. Transcult Psychiatry.

[56] Strauss A, Corbin J (1997). Grounded theory in practice.

[57] Dedoose [program], Version 7.0.23. Los Angeles, CA: SocioCultural Research Consultants, LLC. 2016.

