# Supplementary material for: Comparing self- and provider-collected swabbing for HPV DNA testing in female-to-male transgender adult patients: a mixed-methods biobehavioral study protocol
Source: BMC Infect Dis. 2017 Jun 23;17:444. doi: 10.1186/s12879-017-2539-x (PMC5481878; doi:10.1186/s12879-017-2539-x)
Supplement: Supplementary file 1 — Diagram & Definition of Terms. (PDF 311 kb) [file 12879_2017_2539_MOESM1_ESM.pdf]

Diagram & Definition of Terms

People do not all use the same words or names to talk about their body parts. This makes it challenging for us to give clear self-swab instructions while being sensitive to individuals' identities and preferences. Throughout this study, we use the word **"frontal canal"** to replace the word vagina or vaginal canal. The word **"rectum"** is used to indicate anus or butt hole. We realized that these may not be the words you use. However, it is important for this research project that we use words that are clear and consistent so that everyone understands what body part we are referring to.

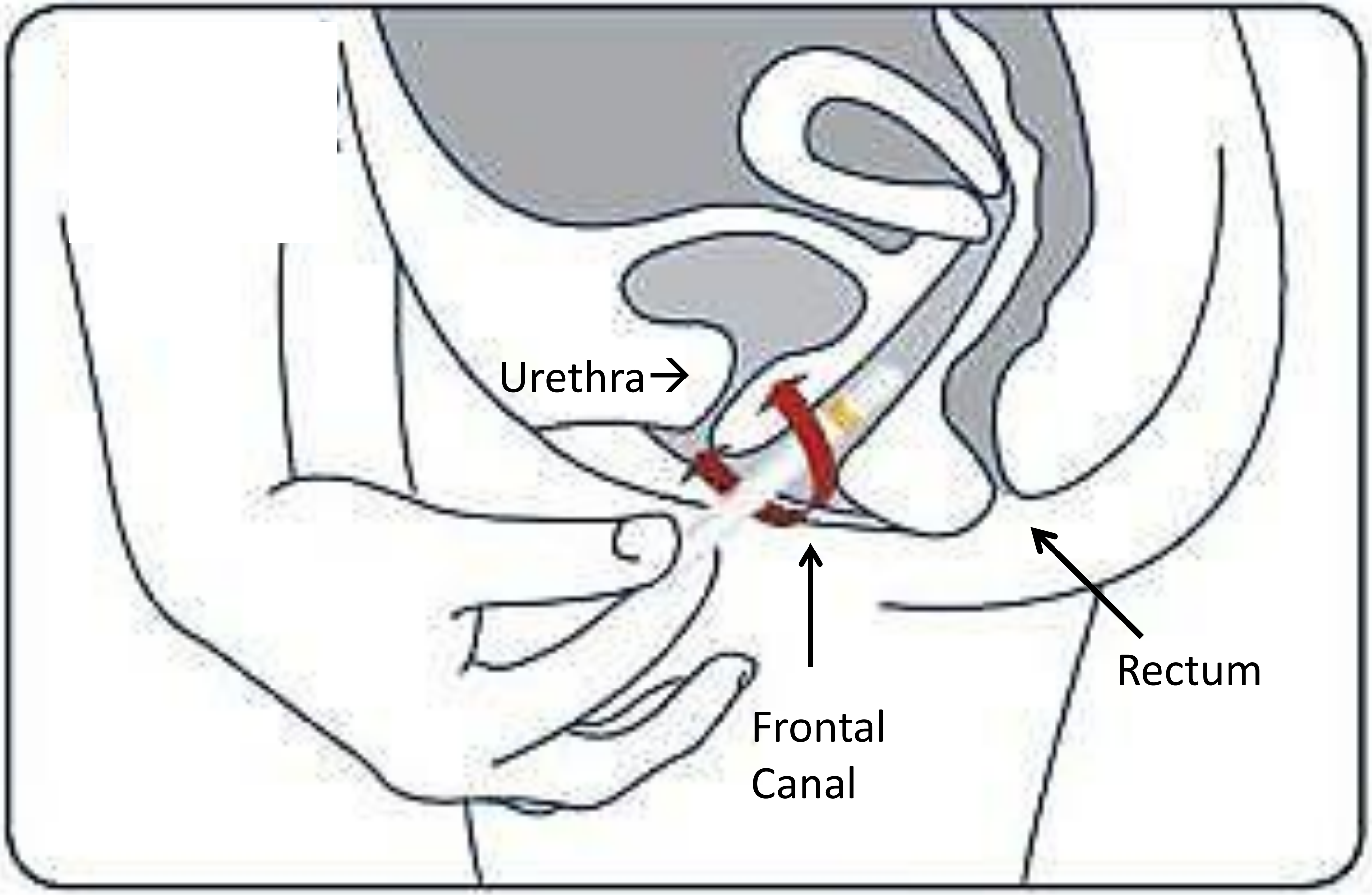

Step 1: Self-Collection of Frontal GC/CT Specimen

# of swabs: 1

Undress from the waist down. Wash and dry hands before beginning. Comfortably position yourself so that you will maintain your balance throughout collection. Open the kit with the **RED** dot. Set the tube aside.

1. Open envelope opposite the cotton tip. Remove swab. Do not touch the cotton end or lay the swab down. (**NOTE:** If you touch the swab tip, drop the swab, or the swab is laid down, discard the swab and request a new one.)
2. Hold swab shaft between thumb and forefinger.
3. Insert the soft tip into the frontal canal approximately 2 inches (see line opposite for reference). Move the swab slowly in a circular motion for **10-30 seconds**, touching all walls of the frontal canal. Withdraw the swab carefully. Avoid touching the surrounding skin.
4. Unscrew the cap from the tube.
5. Immediately place the swab into the tube, cotton end down.
6. Break the swab shaft at the scoreline (dented line around middle of stick). The cotton end will remain in the tube.
7. Discard the top portion of the swab shaft.
8. Tightly screw the cap onto the tube .
9. Place the tube in the small plastic bag with the **RED** dot and seal the bag.
10. Place the sample in the paper bag provided to you.
11. Wash and dry hands.

←This line is 2 inches→

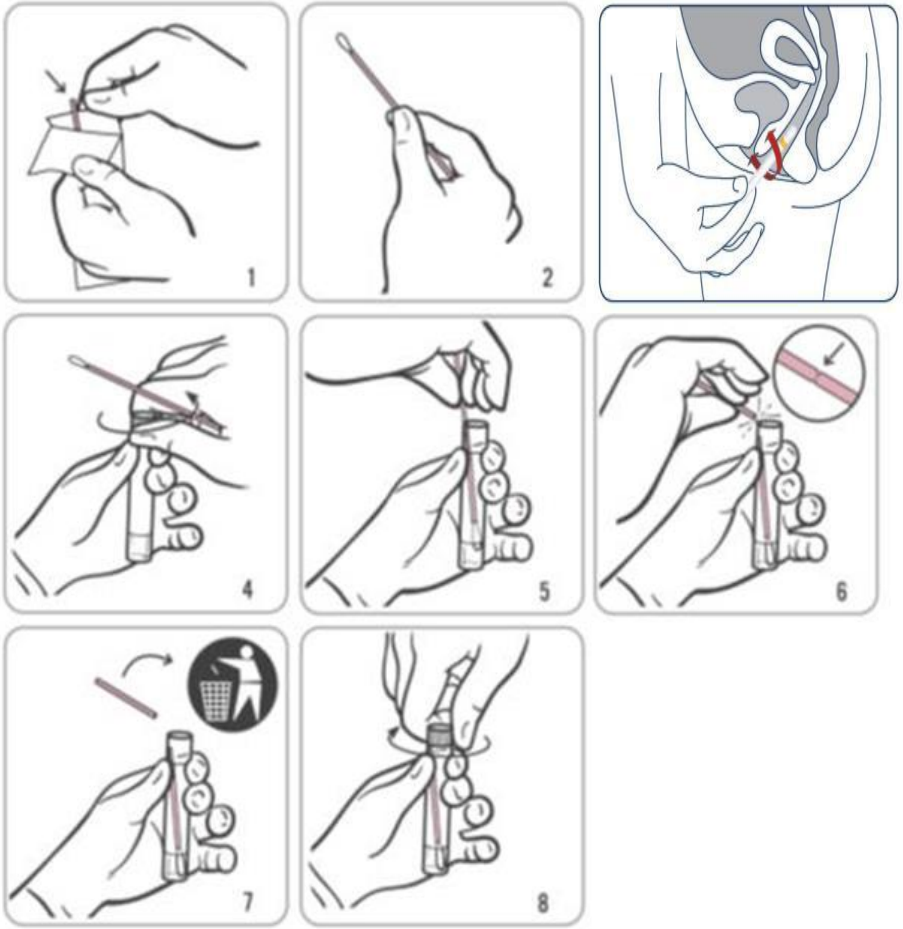

Adapted from: Gen-Probe APTIMA V. Swab Specimen Collection Kit Instructions
MTN-020 Self-collected V. Swab Instructions 30Oct2012
NC Sexually Transmitted Diseases Public Health Public Health Program Manual/Laboratory Testing & Standing Orders Self-Collected Swabs April 2011

Step 2: Self-Collection of Frontal HPV Specimen

# of swabs: 1

Comfortably position yourself so that you will maintain your balance throughout collection.

1. Open the swab with the **BLUE** dot, opposite the cotton tip. Remove swab. Do not touch the cotton end or lay the swab down. (**NOTE:** If you touch the swab tip, drop the swab, or the swab is laid down, discard the swab and request a new one.)
2. Hold swab shaft between thumb and forefinger.
3. Insert the soft tip into the frontal canal approximately 2 inches (see line opposite for reference). Move the swab slowly in a circular motion for **10-30 seconds**, touching all walls of the frontal canal. Withdraw the swab carefully. Avoid touching the surrounding skin.
4. Unscrew the cap from the ThinPrep canister.
5. Immediately place the swab into the canister, cotton end down.
6. Swish the swab in the liquid inside the tube for 10 seconds.
7. Discard the swab in the trash can.
8. Tightly screw the cap back onto the canister.
9. Place the canister in the small plastic bag with the **BLUE** dot and seal the bag.
10. Place the sample in the paper bag provided to you.
11. Wash and dry hands.

←This line is 2 inches→

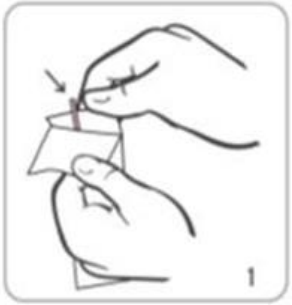

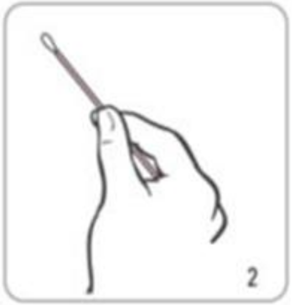

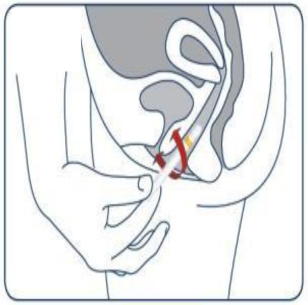

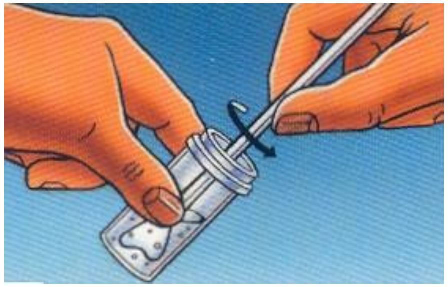

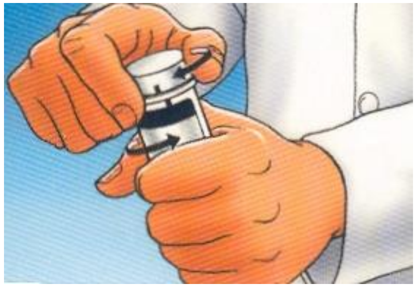

Adapted from: 1). Gen-Probe APTIMA V. Swab Specimen Collection Kit Instructions
MTN-020 Self-collected V. Swab Instructions 30Oct2012
NC Sexually Transmitted Diseases Public Health Public Health Program Manual/Laboratory Testing & Standing Orders Self-Collected Swabs April 2011
2.) Thin Prep Pap Brush/Spatula Protocol. Hologic Inc. 2010.

Step 3: Self-Collection of Rectal GC/CT Specimen

# of swabs: 1

Comfortably position yourself so that you will maintain your balance throughout collection. (Note: Some people find it easier to lay on their side on an exam table with their knees bent ("fetal position").

1. Open envelope with the GREEN dot, opposite the cotton tip. Set the tube aside. Remove swab. Do not touch the cotton end or lay the swab down. (**NOTE:** If you touch the swab tip, drop the swab, or the swab is laid down, discard the swab and request a new one.)
2. Insert the soft tip into the rectum approximately 1 inch.

←This line is 1 inch→

3. Rotate the swab against the rectal wall **at least 3 times**. Withdraw the swab carefully. Avoid touching the surrounding skin.
4. Unscrew the cap from the tube.
5. Immediately place the swab into the tube, cotton end down.
6. Break the swab shaft at the scoreline (dented line around middle of stick). The cotton end will remain in the tube.
7. Discard the top portion of the swab shaft.
8. Tightly screw the cap back onto the tube .
9. Place the tube in the small plastic bag with the GREEN dot.
10. Place the sample in the paper bag provided to you.
11. Wash and dry hands.

You're all done! Once you are dressed, bring the paper bag with the 3 samples to the study Research Assistant, who will label the samples.
