# Supplementary material for: Comparing self- and provider-collected swabbing for HPV DNA testing in female-to-male transgender adult patients: a mixed-methods biobehavioral study protocol
Source: BMC Infect Dis. 2017 Jun 23;17:444. doi: 10.1186/s12879-017-2539-x (PMC5481878; doi:10.1186/s12879-017-2539-x)
Supplement: Supplementary file 2 — Provider Assessment and Interaction Tool. (DOCX 34 kb) [file 12879_2017_2539_MOESM2_ESM.docx]

**Additional file 2: Provider Assessment and Interaction Tool**

**Prior to Physical Exam**

**Height:** inches **Weight:** lbs

**I. GENDER AFFIRMATION**

**What are your preferred pronouns?**

| - He/Him - She/Her - They/Them - Other Pronouns; Specify: | - No Pronoun Preference - Prefers No Pronouns Are Used - Not asked |
| --- | --- |

**Are you taking cross-gender hormones?**

- Yes
- No
- Not asked
- Preferred not to answer

Describe Type/Dose/Frequency (probe prescribed & non-prescribed):

**How long have you been on hormones?**

| - Less than 6 months - 6 months to less than 12 months (6 mo - < 1yr) - 12 months to less than 18 months (1 yr - < 1.5 yrs) - 18 months to less than 24 months (1.5 yrs - < 2 yrs) - 24 months to less than 30 months (2 yrs - < 2.5 yrs) - 30 months to less than 36 months (2.5 yrs - < 3 yrs) - 36 months to less than 42 months (3 yrs - < 3.5 yrs | - 42 months to 48 months (3.5 yrs - < 4 yrs) - 48 months to 54 months (4 yrs - < 4.5 yrs) - 54 months to 60 months (4.5 yrs - < 5 yrs) - 60 months to 66 months (5 yrs - < 5.5 yrs) - 66 months to 72 months (5.5 yrs - < 6 yrs) - 72 months to 78 months (6 yrs - < 6.5 yrs) - 78 months or more (6.5 yrs or longer) |
| --- | --- |

**Have you had any gender confirming surgeries or procedures?**

- Yes; Describe:
- No
- Not asked
- Preferred not to answer

**How long ago did you have these procedures**?

**Do you have preferred language that you use to refer to your body (i.e., genitals)?**

- Yes; Describe:
- No
- Not asked

**II. GENERAL HEALTH HISTORY**

**Have you ever had a cervical Pap test?**

- Yes
- No
- Not asked
- Preferred not to answer

**How long ago was your last Pap test?**

| - A year ago or less - More than 1 year ago but not more than 2 years - More than 2 years ago but not more than 3 years - More than 3 years ago but not more than 5 years | - More than 5 years ago but not more than 7 years - More than 7 years ago but not more than 9 years - More than 9 years ago - I don’t know |
| --- | --- |

**Have you ever had an inadequate cervical Pap test? (Where the doctor could not get a sufficient sample on the swab and you had to be re-tested?)**

- Yes
- No
- I don’t know/ I’m not sure

**Have you ever had an abnormal cervical Pap test?**

- Yes
- No
- Not asked
- Preferred not to answer

**Have you ever had a colposcopy due to an abnormal Pap test?**

- Yes
- No
- Don’t know/Not sure
- Not asked
- Preferred not to answer

**Have you ever had a surgical procedure (LEEP, cone biopsy) due to an abnormal cervical Pap test?**

- Yes
- No
- I don’t know/ I’m not sure

**Have you ever had a test for Human Papillomavirus (HPV)?**

- Yes
- No
- I don’t know/ I’m not sure

**Have you ever had cervical HPV diagnosed by a provider?**

- Yes
- No
- I don’t know/ I’m not sure

**III. SEXUAL HEALTH HISTORY**

Script for Clinical Interview of Sexual Risk - The 5 “P”s of sexual health:

1. Partners
2. Practices
3. Protection from STIs
4. Past history of STIs
5. Prevention of pregnancy
6. Desire and Satisfaction
7. Abuse & IPV

**Opening the Conversation**

*At this point, I’m going to ask you some pretty detailed questions about your sexual health and sexual practices. I understand that these topics are very personal. We ask about this so that we can better understand your risk for sexually transmitted infections (STIs) and also how your sexuality might contribute to your overall health and well-being. These questions also give you an opportunity to address any questions or concerns you might have about sex and your sexual practices.*

*We are also asking these questions because part of our aim is to develop best clinical practices for talking to trans masculine individuals about their sexual health. These questions will help us to determine guidelines to improve care for trans masculine individuals and to better instruct other providers.*

**P1. Partners**

| **Provider Questions** | **Participant Responses** |
| --- | --- |
| Are you currently in a sexual or romantic relationship with anyone? If yes, is this an exclusive relationship? Or are you having sex with anyone else?   - If no, how long ago was your last sexual relationship?   **In the past 12 months**, how many partners have you had? This can be an approximate number if you have had many partners.  How would your partners identify themselves in terms of gender?   - If trans… Tell me about the kind of genitals your partner(s) have (i.e., have they had any type of genital surgery?). |  |

**P2. Practices**

*Now I’m going to ask some more direct/specific questions about your sexual practices. This helps me to understand and speak with you about your risk for STIs and other sexual health issues.*

| **Provider Questions** | **Participant Responses** |
| --- | --- |
| **In the past 12 months,** when having sex…  Do any of your partners touch or have contact with your *[insert response to question above about preferred language for genitals]* ?  Do you have any kind of penetrative sex, taking a penis or fingers into your *[insert response to question above about preferred language for genitals]* ?   - Do you experience discomfort or pain during or after penetration? - Do you have any bleeding after penetration?   Do you have sex with you and your partners genitals rubbing together?  Do you have oral sex?   - If yes…With your mouth on your partner’s genitals? Their mouth on your *[insert response to question above about preferred language for genitals]*?   Do you have anal sex, any contact with your anal area or your partner’s anal area?   - If yes… With genitals, mouth, fingers? Does anything ever go inside your anus?   Do you use toys (dildoes or vibrators) inside your *[insert response to question above about preferred language for genitals]* or anus, or do you use them on your partners?  Do you have any other types of sex that hasn’t been asked about? (e.g., fisting, urethral manipulation/sounding, BDSM, kink, etc.) |  |

**P3. Protection from STIs**

| **Provider Questions** | **Participant Responses** |
| --- | --- |
| What do you do to try to protect yourself and your partners from sexually transmitted infections? Do you use any kind of “protection”, like condoms or other barriers?  Are there some kinds of sex where you do not use barriers? Why?  **In the past 12 months,** how often did you use protection of some kind…always, sometimes, never?  How do you decide when or when not to use protection?  Do you feel that you can insist on using barriers and other methods to prevent STIs or unplanned pregnancies? With some/all/none of your sexual encounters?  **In the past 12 months,** have you had any sexual activity that you think might put you at risk for STIs or HIV?  Do you have any other questions, or are there other forms of protection from STIs that you would like to discuss today? |  |

**P4. Past history of STIs**

| **Provider Questions** | **Participant Responses** |
| --- | --- |
| Do you know if you have you ever been tested for any Sexually Transmitted Infections or HIV? When was the last time that you were tested?  Have you ever been diagnosed with an STI?   - If yes… Do you remember the site of the STI? *[insert response to question above about preferred language for genitals]* Frontal, rectal, throat? - Do you remember how you were treated for the STI? - Have you had any symptoms after treatment?   Are you having any symptoms of an STI now? Such as burning with urination, any unusual discharge, any unusual sore throat or anal pain, any sores or bumps in the genital area, the anal area or inside your mouth?  **In the past 12 months,** do you know if any of your partners have been tested for STIs or HIV?  If they were diagnosed, did you also get tested or treated? |  |

**P5. Pregnancy**

| **Provider Questions** | **Participant Responses** |
| --- | --- |
| How old were you when you experienced your first period (i.e., onset of menstruation)?  How long ago was the last time you had any form of genital bleeding (including spotting)?  Have you ever used any forms of birth control to prevent pregnancy? If so, what forms have you used?  Do you use any kind of birth control now? Would you like more information about birth control?  Have you ever been pregnant?   - If yes… How many times? Have you had any deliveries? Were they natural deliveries or C-sections?   Have you considered having a child of your own that you would carry? Have you considered utilizing a surrogate with your egg? |  |

*Now I’m going to ask a few questions about your sexual desire and satisfaction.*

**6. Sexual Desire & Satisfaction**

| **Provider Questions** | **Participant Responses** |
| --- | --- |
| Do you feel you are able to become physically aroused during sex, such as becoming wet or hard?  How satisfied are you with your ability to achieve orgasm? Do you have any pain during or after orgasm?  How satisfied are you with your overall sex life? |  |

**7. Sexual Abuse & IPV**

| **Provider Questions** | **Participant Responses** |
| --- | --- |
| Has anyone ever forced or compelled you to do anything sexually that you did not want to do?   - Is this something that is currently happening, or in the past? - If in the past… how long ago did this happen? - Would you like to speak with anyone here about this, or get additional resources?   ** *If participant endorses current or past abuse, please check in to make sure they still feel comfortable proceeding with the exam.* |  |

**Closing the Conversation**

| **Provider Questions** | **Participant Responses** |
| --- | --- |
| Do you have any other concerns or questions about your sexual health or sexual practices?  Is there anything else you think is important for me to know about your overall sexual health? |  |

**V. EXPLANATION OF PAP PROCEDURES**

**PROVIDER: Please explain the swabbing and Pap testing procedures to the participant. Then ask the following questions prior to leaving the room while the participant undresses.**

Provider Script:

In a moment, I’ll step out to let you get ready for the examination. Before I do that, I wanted to make sure that you understand the examination.

The Pap test is a test for cervical cancer. The cervix is the opening to the uterus, and it is a fairly common place for cancer to develop. In fact, what we are looking for are cells from the cervix which may look abnormal and might be a sign of a cancer developing so that we can monitor closely or treat the abnormal cells in order to prevent cervical cancer. So, I’ll be basically brushing the outside of the cervix and brushing the inside of the cervix in order to obtain cells that we will put in a liquid and send to the pathologist to examine.

Because we know that infection with the HPV virus can trigger cells to start growing abnormally and possibly turn into cancer, we sometimes also will test for HPV along with the Pap test. We’ll be testing you for HPV today. One of the self-swabs that you are doing is also a test for HPV.

We’ll also do three swabs of the genital tract/front hole to check for common infections: gonorrhea, chlamydia, trichomonas and bacterial vaginosis. We’ll help you arrange for treatment if we find any infection.

Traditionally, when a pelvic and pap smear is done, the patient lays on the examination table with their feet up in stirrups, but I want you to know that you can choose something other than this. Some people prefer to have their feet flat on the end of the table; other people lay with the soles of their feet together and their legs out in a frog-like position. Some people would prefer to have the top of the exam table raised up a bit so that they can better see what is going on during the examination.

**How would you like to be positioned for the examination?**

Some people also find that they have discomfort when the speculum is inserted and that it helps them to insert the speculum themselves with my guidance.

**Is this something that you would like to do or try?**

**Would it be helpful to you to have someone else, like a friend or an assistant, in the room to talk to or to be with you while you are having the examination?**

**Before we do the exam, would you like to see the speculum and the swabs and brushes that we use?**

**Would you like me to talk you through what I’m doing as the exam goes along or would you prefer that I remain silent?**

Finally, I want you to know that you are in control of the examination. If at any point, you would like me to pause or stop the examination for any reason, just let me know. You can tell me to stop or simply hold up your hand, and I’ll check in with you about what you want to do.

**VI. PATIENT READINESS**

[PROVIDER: Please answer the following questions. DO NOT ask them of the patient.]

**How would you rate the patient’s knowledge and understanding of their own health?**

| 1 | 2 | 3 | 4 |
| --- | --- | --- | --- |
| Poor | Fair | Good | Excellent |

**How would you rate this patient’s level of self-advocacy?**

| 1 | 2 | 3 | 4 |
| --- | --- | --- | --- |
| Poor | Fair | Good | Excellent |

**How anxious does the patient seem to be?**

| 1 | 2 | 3 | 4 |
| --- | --- | --- | --- |
| Not at all anxious | Mildly anxious | Very anxious | Extremely anxious |

**How anxious do you feel about conducting the exam?**

| 1 | 2 | 3 | 4 |
| --- | --- | --- | --- |
| Not at all anxious | Mildly anxious | Very anxious | Extremely anxious |

**Immediately Following the Physical Exam**

[PROVIDER: Please answer the following questions before conducting the exam. DO NOT ask them of the patient.]

**Please rate the patient’s level emotional comfort during the exam.**

| 1 | 2 | 3 | 4 |
| --- | --- | --- | --- |
| Very Uncomfortable | Uncomfortable | Comfortable | Very Comfortable |

**Please rate the patient’s level of physical pain or discomfort during the exam.**

| 1 | 2 | 3 | 4 |
| --- | --- | --- | --- |
| Very Uncomfortable | Uncomfortable | Comfortable | Very Comfortable |

**Please rate the number of questions/concerns the patient had prior to the exam.**

| 1 | 2 | 3 | 4 |
| --- | --- | --- | --- |
| None | Few | A lot | Excessive |

**How well do you feel you answered the patient’s questions and addressed their concerns?**

| 1 | 2 | 3 | 4 |
| --- | --- | --- | --- |
| Not Well | Fairly Well | Well | Very Well |

**How satisfied are you with how well you put the patient at ease in this clinical encounter?**

| 1 | 2 | 3 | 4 |
| --- | --- | --- | --- |
| Very Dissatisfied | Dissatisfied | Satisfied | Very Satisfied |

**Please rate your own level of comfort during this encounter.**

| 1 | 2 | 3 | 4 |
| --- | --- | --- | --- |
| Very Uncomfortable | Uncomfortable | Comfortable | Very Comfortable |

**Please rate your own level of comfort after performing the exam.**

| 1 | 2 | 3 | 4 |
| --- | --- | --- | --- |
| Very Uncomfortable | Uncomfortable | Comfortable | Very Comfortable |

**Overall, how satisfied are you with your interaction with the participant in this clinical encounter?**

| 1 | 2 | 3 | 4 |
| --- | --- | --- | --- |
| Very Dissatisfied | Dissatisfied | Satisfied | Very Satisfied |

**TECHNICAL CONSIDERATIONS:**

**What size speculum was used with this patient?**

- Large
- Medium
- Small
- Pediatric

**Was lubricant gel used?**

- Yes
- No

**How difficult or easy was speculum insertion?**

| 1 | 2 | 3 | 4 |
| --- | --- | --- | --- |
| Very Difficult | Difficult | Easy | Very Easy |

**How easy or difficult was it for you to visualize the cervix?**

| 1 | 2 | 3 | 4 |
| --- | --- | --- | --- |
| Very Difficult  (Blind Swabbing) | Difficult | Easy | Very Easy(Well Visualized) |

**Blood:**

| 1 | 2 | 3 | 4 |
| --- | --- | --- | --- |
| None | Scant | Moderate | Obscuring |

**Discharge:**

| 1 | 2 | 3 | 4 |
| --- | --- | --- | --- |
| None | Scant | Moderate | Obscuring |

**Accommodations made during the exam:**

- No accommodations
- Chaperone; Specify relationship to participant:
- Positioning during exam (on side, feet flat on table):
- Participant self-insertion of speculum
- Insertion of speculum by partner
- Other accommodation; Specify:

**Please estimate the time spent performing the pelvic exam/Pap test: minutes**

**Please estimate the total amount of time you spent with the patient:** **minutes**

**Other comments or remarks about the clinical encounter?**
