# Supplementary material for: Comparing self- and provider-collected swabbing for HPV DNA testing in female-to-male transgender adult patients: a mixed-methods biobehavioral study protocol
Source: BMC Infect Dis. 2017 Jun 23;17:444. doi: 10.1186/s12879-017-2539-x (PMC5481878; doi:10.1186/s12879-017-2539-x)
Supplement: Supplementary file 3 — Patient Satisfaction Survey. (DOC 48 kb) [file 12879_2017_2539_MOESM3_ESM.doc]

Additional file 3:

Patient Satisfaction Survey

*As we discussed during the informed consent process, part of today’s visit is a reflection on the interaction you had with the provider. Remember that your responses will be kept anonymous; your provider will not be able to see your name or connect your responses to who you are. We will not share your responses with the provider until a later date. Please be as honest as possible. We also welcome your comments and suggestions at the end. Thank you! We appreciate your participation.*

PARTICIPANT COMFORT AND SATISFACTION

Please help us know how we are doing by answering some questions about the services you have received.

1. How would you rate your overall level of emotional discomfort during the exam?

| 1 | 2 | 3 | 4 |
| --- | --- | --- | --- |
| Very Uncomfortable | Uncomfortable | Comfortable | Very Comfortable |

1. How would you rate your overall level of physical pain or discomfort during the provider exam?

0 1 2 3 4 5 6 7 8 9 10

No Moderate Worst

Pain Pain Pain

Possible

1. How comfortable were you with asking the provider questions during the procedure?

| 1 | 2 | 3 | 4 |
| --- | --- | --- | --- |
| Very Uncomfortable | Uncomfortable | Comfortable | Very Comfortable |

1. How comfortable were you with the procedures being performed?

| 1 | 2 | 3 | 4 |
| --- | --- | --- | --- |
| Very Uncomfortable | Uncomfortable | Comfortable | Very Comfortable |

1. Overall, how satisfied are you with the courtesy and respect you were given?

| 1 | 2 | 3 | 4 |
| --- | --- | --- | --- |
| Very Dissatisfied | Dissatisfied | Satisfied | Very Satisfied |

1. How satisfied are you with the respect given to your identity, preferred pronoun, and sexual orientation and practices?

| 1 | 2 | 3 | 4 |
| --- | --- | --- | --- |
| Very Dissatisfied | Dissatisfied | Satisfied | Very Satisfied |

1. Do you agree that the provider was knowledgeable about the sexual health needs of trans masculine individuals?

| 1 | 2 | 3 | 4 |
| --- | --- | --- | --- |
| Strongly Disagree | Disagree | Agree | Strongly Agree |

1. How satisfied are you with the provider’s ability to ease any of your anxiety or worry about the procedure? (i.e. answer questions, explain tests/ procedures, etc.)

| 1 | 2 | 3 | 4 |
| --- | --- | --- | --- |
| Very Dissatisfied | Dissatisfied | Satisfied | Very Satisfied |

1. Do you have any other comments about your experience today that you would like to share?
